# Supplementary figures and images for: MKK6 deficiency promotes cardiac dysfunction through MKK3-p38γ/δ-mTOR hyperactivation
Source: eLife. 2022 Aug 16;11:e75250. doi: 10.7554/eLife.75250 (PMC9381040; doi:10.7554/eLife.75250)

Figure 4 - figure supplement 1A

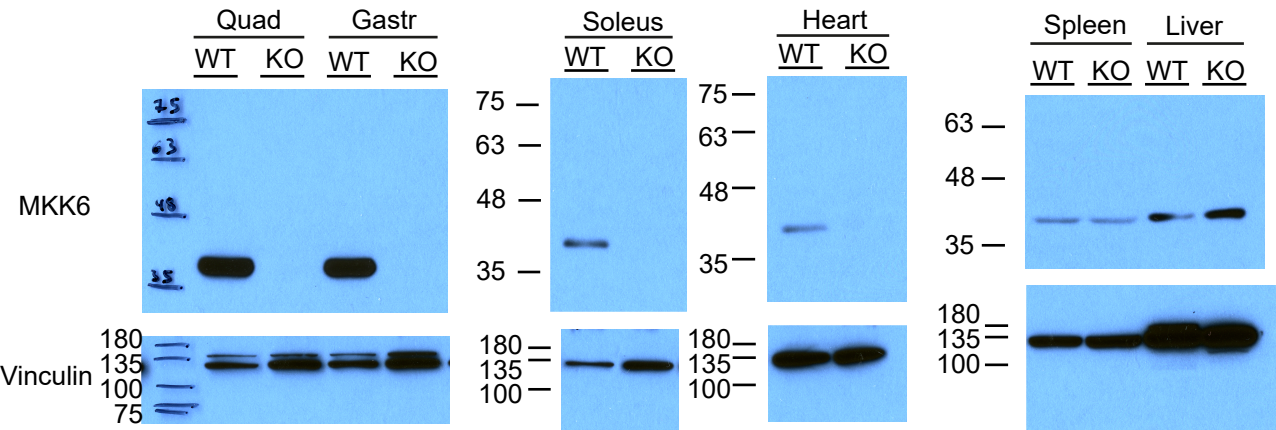

Figure 4 - figure supplement 1B

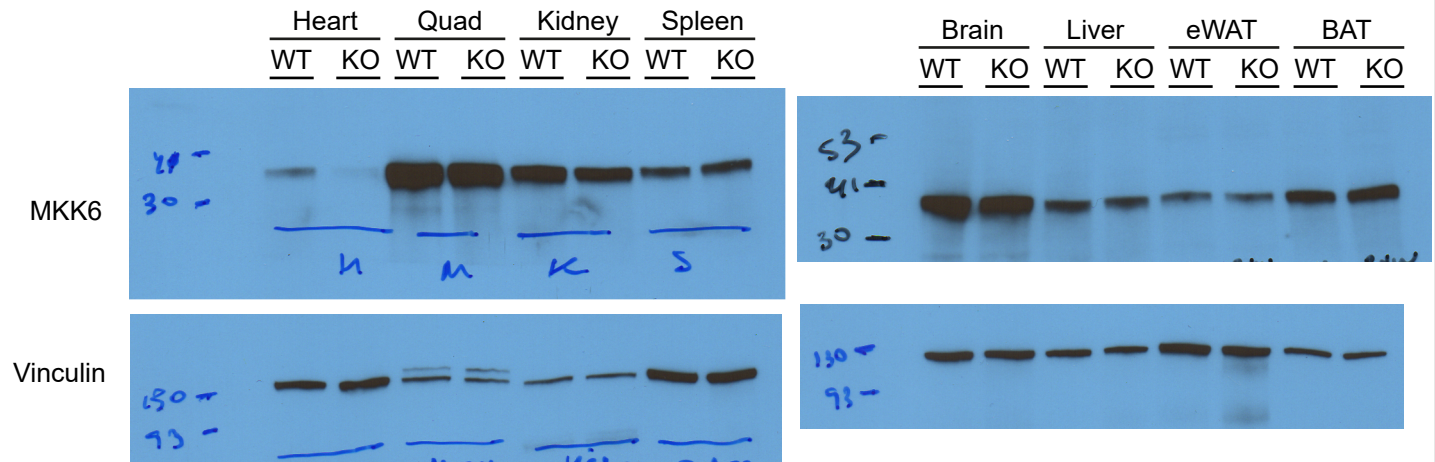

Supplement: Figure 4—figure supplement 1—source data 1. [file elife-75250-fig4-figsupp1-data1.pdf]

**Figure 5A**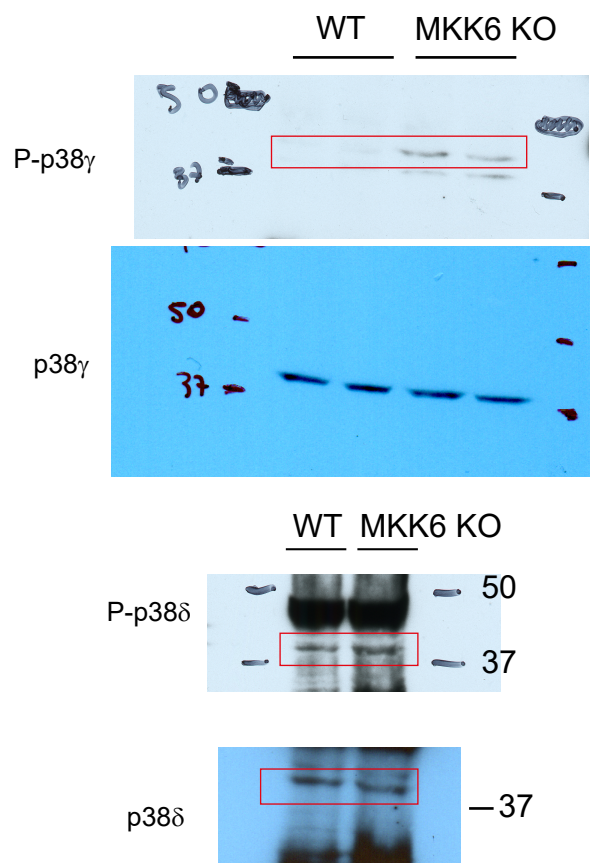**Figure 5B**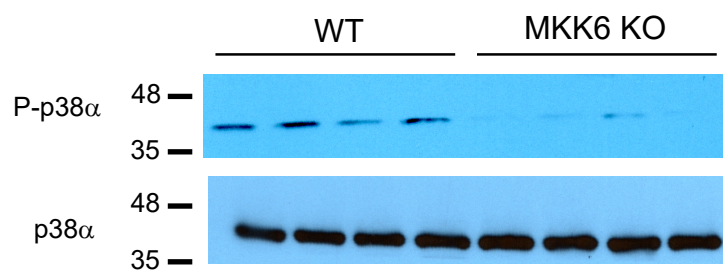**Figure 5C**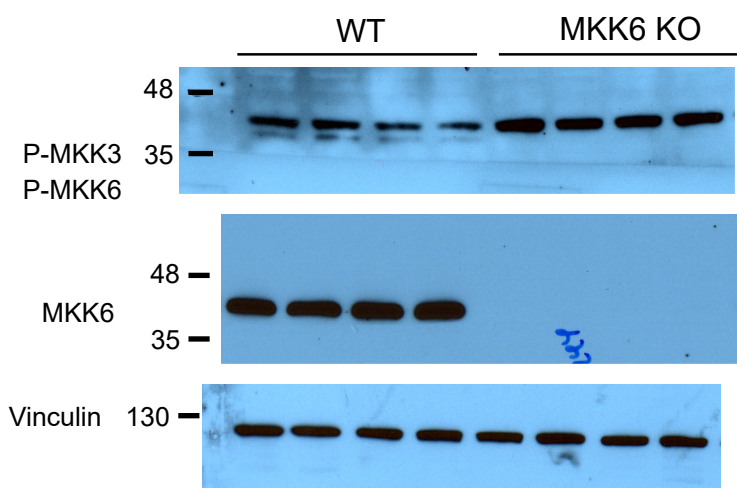**Figure 5C**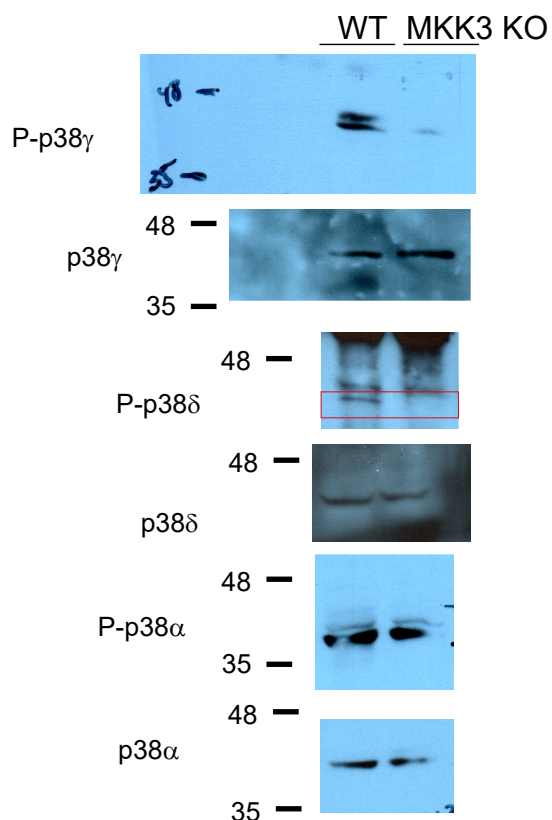

Supplement: Figure 5—source data 1. [file elife-75250-fig5-data1.pdf]

Figure 5 - figure supplement 1A

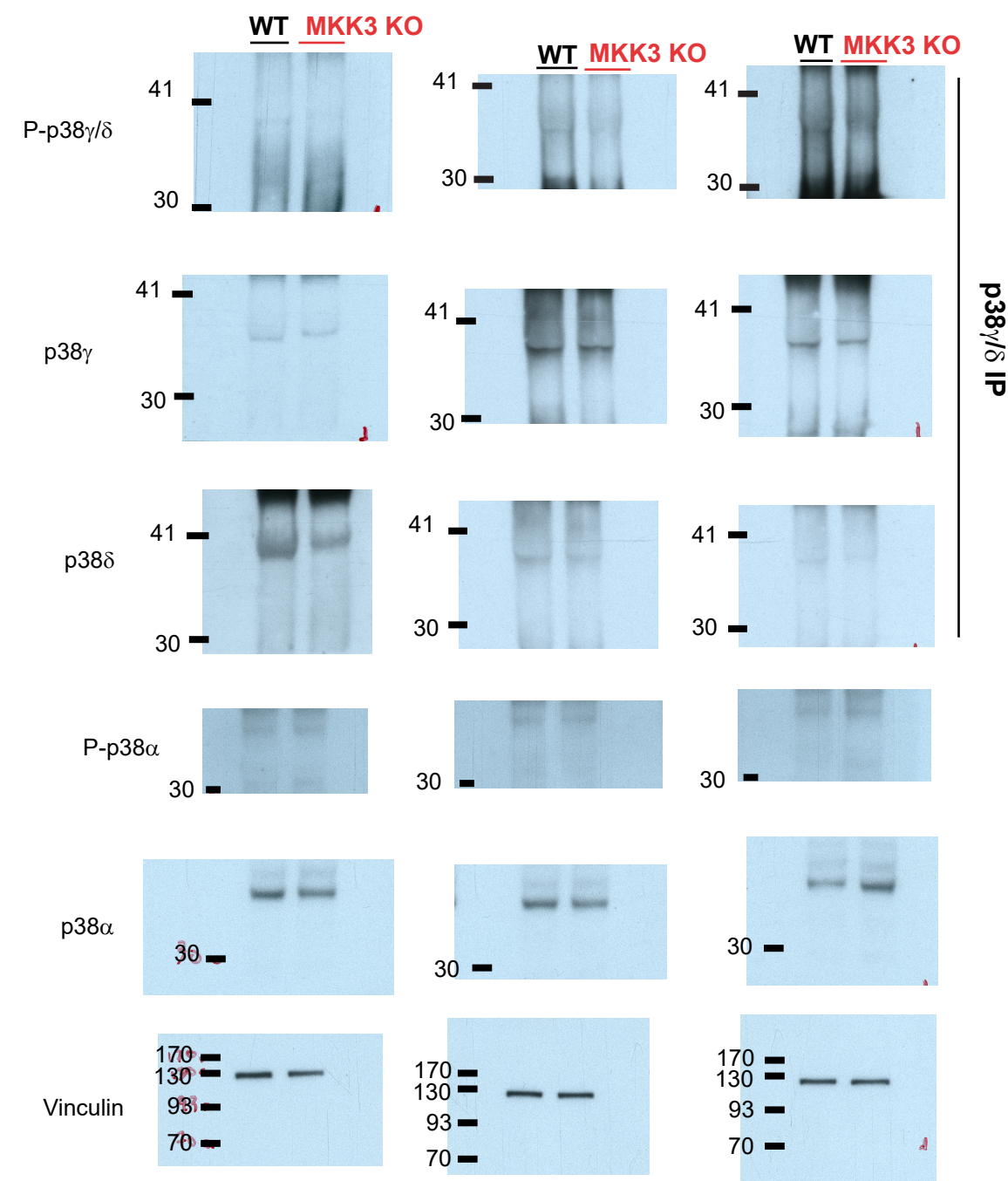

Supplement: Figure 5—figure supplement 1—source data 1. [file elife-75250-fig5-figsupp1-data1.pdf]

Figure 5 - figure supplement 2A

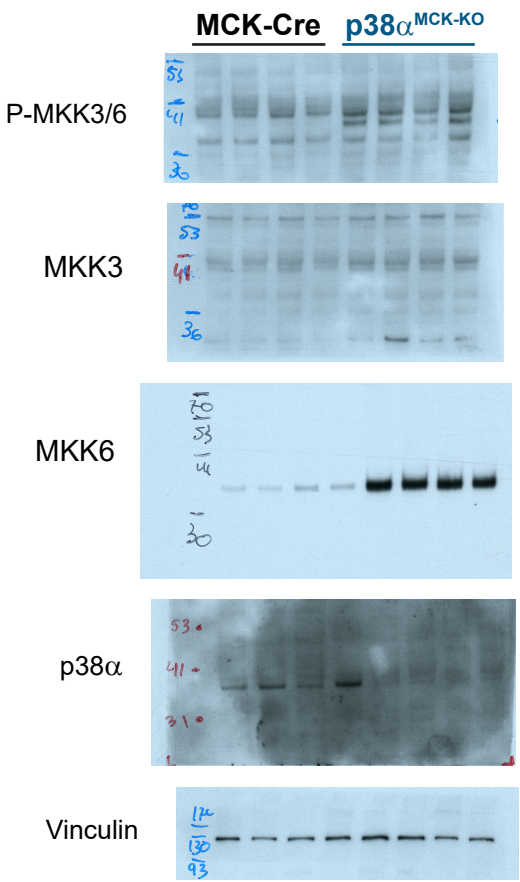

Figure 5 - figure supplement 2C

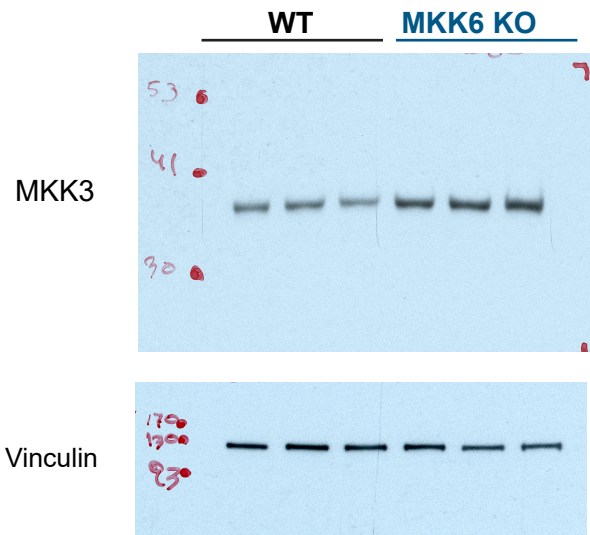

Supplement: Figure 5—figure supplement 2—source data 1. [file elife-75250-fig5-figsupp2-data1.pdf]

# Figure 7A

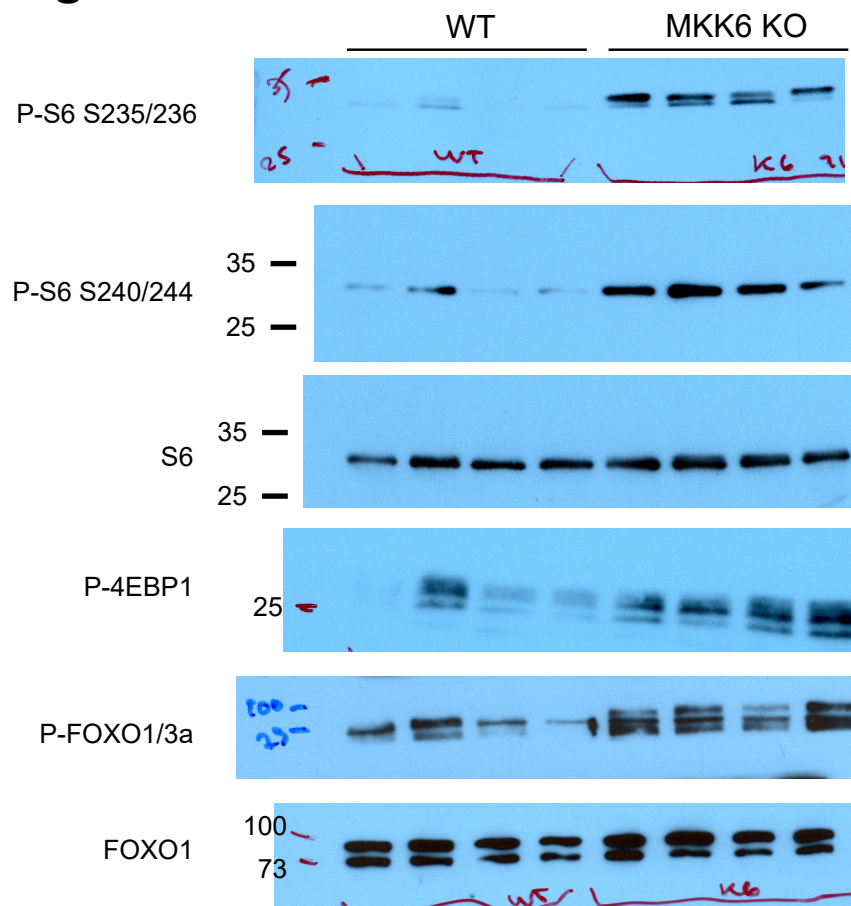

# Figure 7B

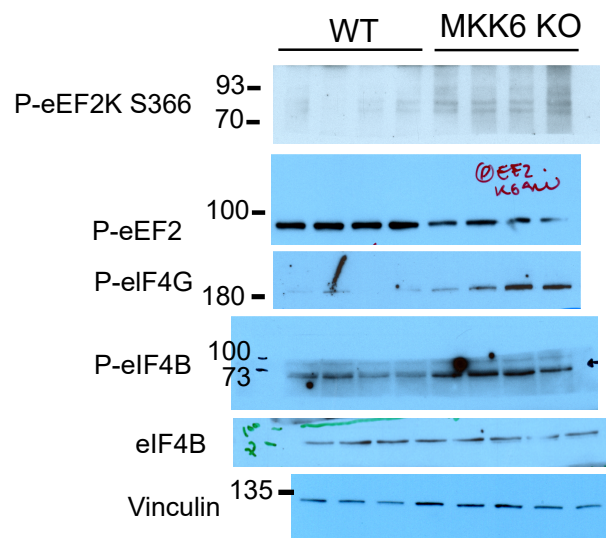

# Figure 7C

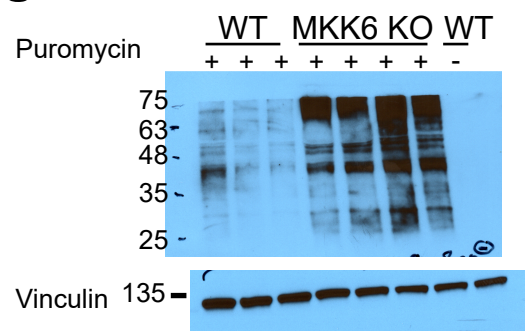

Supplement: Figure 7—source data 1. [file elife-75250-fig7-data1.pdf]

Figure 7 - figure supplement 1A

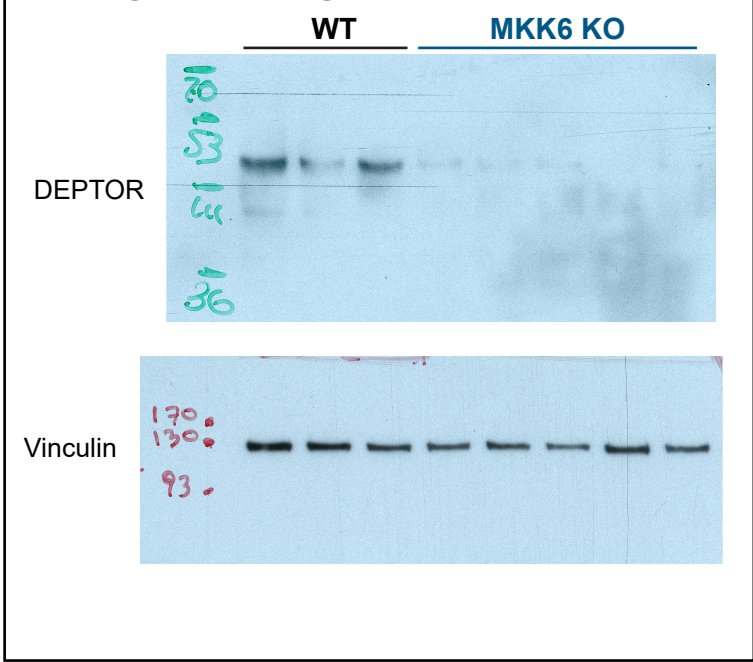

Supplement: Figure 7—figure supplement 1—source data 1. [file elife-75250-fig7-figsupp1-data1.pdf]

**Figure 7 - figure supplement 2B**

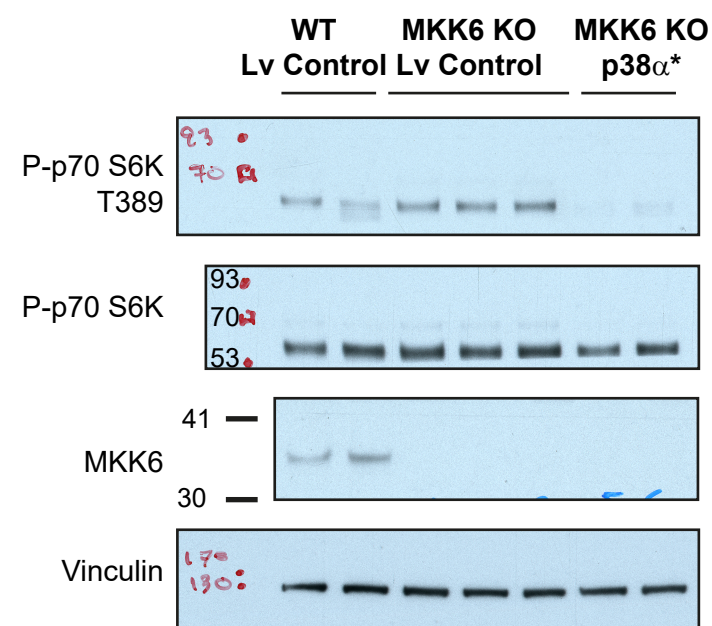

Supplement: Figure 7—figure supplement 2—source data 1. [file elife-75250-fig7-figsupp2-data1.pdf]

Figure 7 - figure supplement 3A

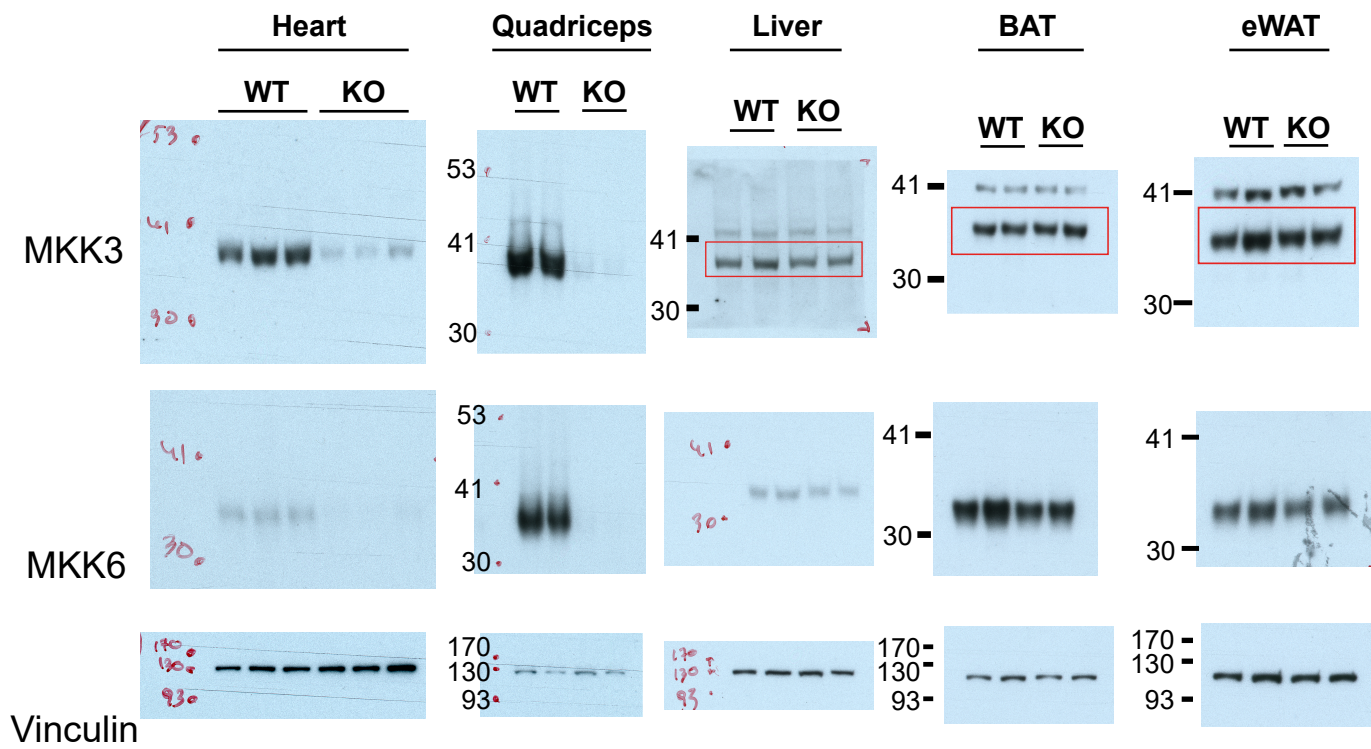

Supplement: Figure 7—figure supplement 3—source data 1. [file elife-75250-fig7-figsupp3-data1.pdf]

Figure 7 - figure supplement 4A

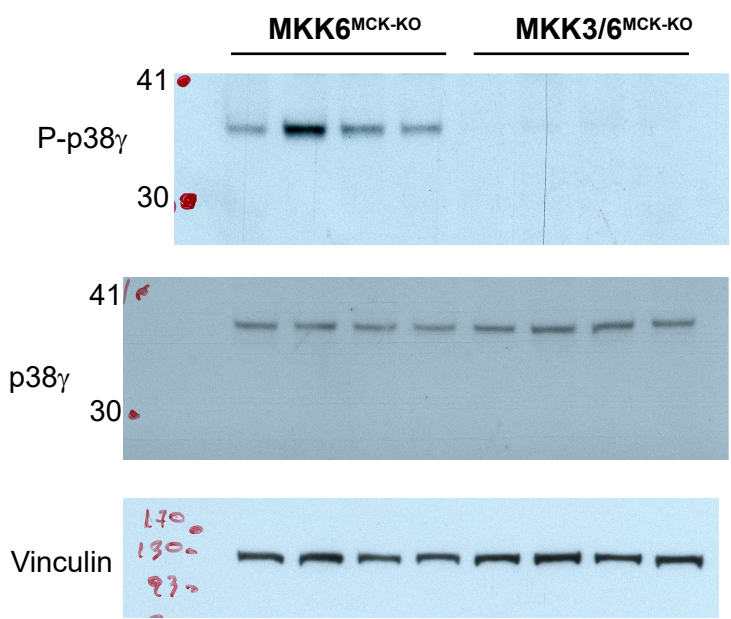

Figure 7 - figure supplement 4B

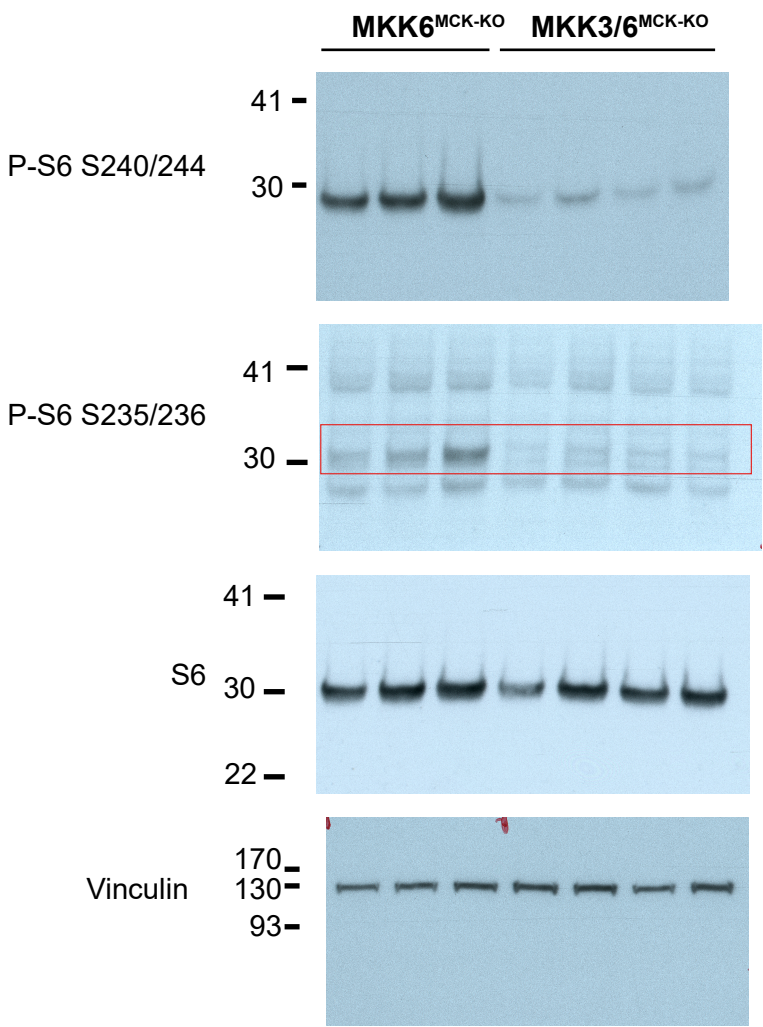

Supplement: Figure 7—figure supplement 4—source data 1. [file elife-75250-fig7-figsupp4-data1.pdf]
