# Supplementary material for: MKK6 deficiency promotes cardiac dysfunction through MKK3-p38γ/δ-mTOR hyperactivation
Source: eLife. 2022 Aug 16;11:e75250. doi: 10.7554/eLife.75250 (PMC9381040; doi:10.7554/eLife.75250)
Supplement: Transparent reporting form [file elife-75250-transrepform1.docx]

***eLife’s* transparent reporting form**

We encourage authors to provide detailed information *within their submission* to facilitate the interpretation and replication of experiments. Authors can upload supporting documentation to indicate the use of appropriate reporting guidelines for health-related research (see [EQUATOR Network](http://www.equator-network.org/%20)), life science research (see the [BioSharing Information Resource](https://biosharing.org/)), or the [ARRIVE guidelines](http://www.plosbiology.org/article/info:doi/10.1371/journal.pbio.1000412) for reporting work involving animal research. Where applicable, authors should refer to any relevant reporting standards documents in this form.

If you have any questions, please consult our Journal Policies and/or contact us: [editorial@elifesciences.org](mailto:editorial@elifesciences.org).

**Sample-size estimation**

- You should state whether an appropriate sample size was computed when the study was being designed
- You should state the statistical method of sample size computation and any required assumptions
- If no explicit power analysis was used, you should describe how you decided what sample (replicate) size (number) to use

To choose the size of the sample we have used the 3R rule to ensure statistical validity and significance with the chosen size.

CNIC has biostatisticians to help in designing our animal experiments. They use the most up-to-date statistical methods to ensure that the correct number of animals will be employed in each experiment. The number of animals in each group is determined by the statistical power that is required to detect significant biologically relevant differences. Meaningful differences in means could be detected using the appropriate test in each case. We use a number of mice per group for at least 80% power in the testing.

The exact sample size and number of replicates are indicated in every figure legend. For animal research and in vitro experiments, data were obtained from at least 2-3 independent experiments with high consistency between samples.

Please outline where this information can be found within the submission (e.g., sections or figure legends), or explain why this information doesn’t apply to your submission:

**Replicates**

- You should report how often each experiment was performed
- You should include a definition of biological versus technical replication
- The data obtained should be provided and sufficient information should be provided to indicate the number of independent biological and/or technical replicates
- If you encountered any outliers, you should describe how these were handled
- Criteria for exclusion/inclusion of data should be clearly stated
- High-throughput sequence data should be uploaded before submission, with a private link for reviewers provided (these are available from both GEO and ArrayExpress)

Each experiment was performed at least 2-3 times to validate its reproducibility. The number of biological replicates is indicated in every figure legend. Animals that presented disease or had been bitten because of fight in the cage were excluded. Robust regression and outlier removal (ROUT) method with a Q (maximum FDR) =1% was applied to identify possible outliers and try to detect whether there was any justifiable reason (such as measurement error) to eliminate them.

Please outline where this information can be found within the submission (e.g., sections or figure legends), or explain why this information doesn’t apply to your submission:

**Statistical reporting**

- Statistical analysis methods should be described and justified
- Raw data should be presented in figures whenever informative to do so (typically when N per group is less than 10)
- For each experiment, you should identify the statistical tests used, exact values of N, definitions of center, methods of multiple test correction, and dispersion and precision measures (e.g., mean, median, SD, SEM, confidence intervals; and, for the major substantive results, a measure of effect size (e.g., Pearson's r, Cohen's d)
- Report exact p-values wherever possible alongside the summary statistics and 95% confidence intervals. These should be reported for all key questions and not only when the p-value is less than 0.05.

Statistical methods and details related to statistical analysis are specified in Materials and Methods section of the main text. Result data are expressed as mean ± SEM. A difference of P<0.05 was considered significant. Gaussian (normal) distribution was determined using the Shapiro-Wilks normality test. For normally distributed populations, differences between groups were examined for statistical significance by two-tailed Student t-test (2 groups) and 1-way ANOVA followed by Tukey post-test (3 or more groups). To test the respective interaction of treatment or age and genotype, a 2-way ANOVA was performed. Tukey or Sidak post-test were subsequently employed when appropriate. For data that failed normality testing, Mann-Whitney test (2 groups), or Kruskal-Wallis with Dunn post-test (3 or more groups) was performed. Gehan-Breslow-Wilcoxon test was used to assess significance in the Kaplan–Meier survival analysis.

Please outline where this information can be found within the submission (e.g., sections or figure legends), or explain why this information doesn’t apply to your submission:

(For large datasets, or papers with a very large number of statistical tests, you may upload a single table file with tests, Ns, etc., with reference to sections in the manuscript.)

**Group allocation**

- Indicate how samples were allocated into experimental groups (in the case of clinical studies, please specify allocation to treatment method); if randomization was used, please also state if restricted randomization was applied
- Indicate if masking was used during group allocation, data collection and/or data analysis

Please outline where this information can be found within the submission (e.g., sections or figure legends), or explain why this information doesn’t apply to your submission:

Animal cages were randomly allocated in our animal facility. Male animals were allocated in experimental groups depending on their genotype and age so experimental groups were balanced in terms of age and number of animals.

Experimenters were blinded analyzing samples except for those experiments (e.g., immunoblots) where samples were needed to be loaded correctly. In the case of *in vivo* experiments, it was not always possible to blind the experimenter to the identity of the animals. To decrease the possibility of introducing a bias in the analysis, each independent experiment was performed by a different investigator when possible.

**Additional data files (“source data”)**

- We encourage you to upload relevant additional data files, such as numerical data that are represented as a graph in a figure, or as a summary table
- Where provided, these should be in the most useful format, and they can be uploaded as “Source data” files linked to a main figure or table
- Include model definition files including the full list of parameters used
- Include code used for data analysis (e.g., R, MatLab)
- Avoid stating that data files are “available upon request”

Please indicate the figures or tables for which source data files have been provided:

Source data is provided for the following figures:

Figure 1

Figure 2

Figure 3

Figure 3 – figure supplement 1

Figure 4

Figure 4 – figure supplement 1

Figure 5

Figure 5 – figure supplement 1

Figure 5 – figure supplement 2

Figure 6

Figure 7

Figure 7 – figure supplement 1
